# *Aedes albopictus* D7 Salivary Protein Prevents Host Hemostasis and Inflammation

**DOI:** 10.3390/biom10101372

**Published:** 2020-09-27

**Authors:** Ines Martin-Martin, Leticia Barion Smith, Andrezza Campos Chagas, Anderson Sá-Nunes, Gaurav Shrivastava, Paola Carolina Valenzuela-Leon, Eric Calvo

**Affiliations:** 1 Laboratory of Malaria and Vector Research, National Institute of Allergy and Infectious Diseases, National Institutes of Health, Rockville, MD 20852, USA; ines.martin-martin@nih.gov (I.M.-M.); leticia.smith@nih.gov (L.B.S.); andrezza.camposchagas.ctr@mail.mil (A.C.C.); gaurav.shrivastava@nih.gov (G.S.); paolacarolina.valenzuelaleon@nih.gov (P.C.V.-L.); 2 Vector & Parasite Biology Department, Entomology Branch. Walter Reed Army Institute of Research, Silver Spring, MD 20910, USA; 3 Department of Immunology, Institute of Biomedical Sciences, University of Sao Paulo, Sao Paulo 05508-000, Brazil; sanunes@usp.br

**Keywords:** mosquito, D7 proteins, arthropods, salivary glands, saliva, isothermal titration calorimetry, platelet aggregation, leukocyte recruitment, leukotrienes, blood feeding

## Abstract

Mosquitoes inject saliva into the host skin to facilitate blood meal acquisition through active compounds that prevent hemostasis. D7 proteins are among the most abundant components of the mosquito saliva and act as scavengers of biogenic amines and eicosanoids. Several members of the D7 family have been characterized at the biochemical level; however, none have been studied thus far in *Aedes albopictus*, a permissive vector for several arboviruses that causes extensive human morbidity and mortality. Here, we report the binding capabilities of a D7 long form protein from *Ae. albopictus* (AlboD7L1) by isothermal titration calorimetry and compared its model structure with previously solved D7 structures. The physiological function of AlboD7L1 was demonstrated by ex vivo platelet aggregation and in vivo leukocyte recruitment experiments. AlboD7L1 binds host hemostasis agonists, including biogenic amines, leukotrienes, and the thromboxane A2 analog U-46619. AlboD7L1 protein model predicts binding of biolipids through its N-terminal domain, while the C-terminal domain binds biogenic amines. We demonstrated the biological function of AlboD7L1 as an inhibitor of both platelet aggregation and cell recruitment of neutrophils and eosinophils. Altogether, this study reinforces the physiological relevance of the D7 salivary proteins as anti-hemostatic and anti-inflammatory molecules that help blood feeding in mosquitoes.

## 1. Introduction

The Asian tiger mosquito, *Aedes albopictus* (Diptera: Culicidae), has undergone a dramatic global expansion starting from Southeast Asia to the Americas, Europe, Africa, and the Middle East [1,2]. *Aedes albopictus* is a highly invasive species due to its ability to withstand long periods of desiccation and persist in colder climates [3]. Moreover, climate change and globalization have contributed to the spread of this mosquito species [4]. *Aedes albopictus* mosquitoes have an aggressive daytime human-biting behavior, and it is able to transmit several arboviruses, including dengue, Zika, Chikungunya, West Nile, or Mayaro viruses [5,6,7].

Female *Ae. albopictus* mosquitoes require vertebrate blood for egg development. This mosquito species is an opportunistic feeder which takes blood meals primarily from mammals, but preferentially feeds on humans [8,9]. *Aedes albopictus* mosquitoes have been reported to feed on domestic and wildlife animals and might serve as bridge vectors by supporting the potential transfer of an acquired pathogen from an infected wild animal to a human host during a subsequent blood-meal [5].

During blood feeding, female mosquitoes insert their mouthparts into the host skin and ingest blood, either directly from a capillary or from the hemorrhagic pool created by the blood leakage [10,11]. This injury triggers activation of host hemostasis, a physiological response to blood loss, and ignites a local inflammatory response. Early local vascular contraction reduces blood flow to the injury site and platelet aggregation forms a soft plug [12,13]. Blood clotting, the secondary step of hemostasis, tightly secures the platelet plug with covalently linked fibrin fibers, thereby providing strength and leading to the formation of a stable clot [14]. Additionally, the host immune system also reacts to the blood vessel and skin injury, resulting in itching and inflammation, characterized by swelling, pain, and redness [15].

Blood-feeding arthropods, by an evolutionary process, have developed an important diversity of pharmacological compounds in their saliva that prevent hemostasis and immune responses in order to counteract these physiological responses from the host [16,17]. Salivary proteins are secreted into the skin during mosquito probing and act as vasodilators, platelet aggregation inhibitors, anticoagulants, and anti-inflammatory molecules [15]. Salivary proteins from *Ae. albopictus* are highly immunogenic and can trigger the generation of IgE and IgG antibodies. *Aedes albopictus* may induce intense local cutaneous reactions and has been reported as the most common species associated with severe systemic allergic reaction to mosquito bites, associated with IgE antibodies [18,19]. Antibodies against *Ae. albopictus* salivary proteins are currently being used as markers to monitor host exposure to specific mosquito bites [20,21,22]. However, only a few studies have been done to identify and characterize the *Ae. albopictus* salivary proteins and much remains unknown.

A transcriptomic analysis of *Ae. albopictus* salivary glands, carried out by Sanger sequencing of a cDNA library, revealed the presence of 69 cDNA’s encoding putative salivary-secreted proteins [23]. This transcriptome consists of the main salivary protein families found in other mosquito species, with high similarities to the *Aedes aegypti* transcriptome [24]. Only a few salivary proteins from *Ae. albopictus* have been biochemically characterized; including an apyrase, as a potent platelet aggregation inhibitor [25,26]; an alpha-glucosidase, present in both male and female salivary glands [25]; a C-type lectin that binds mannose residues [27]; and Alboserpin, a factor Xa inhibitor that serves as a potent anticoagulant [28]. However, to this date, the D7 long forms, one of most studied proteins in salivary glands from insects, have not been biochemically characterized in *Ae. albopictus*.

D7 proteins are distantly related to the odorant-binding family and are among the most abundant components in the salivary glands of several blood feeding arthropods [29,30,31,32,33]. These salivary kratagonists bind and sequester host physiological effectors, inhibiting their hemostatic activities at the bite site [34]. The D7 proteins belong to a multi-gene family that has undergone gene duplication and functional divergence, resulting in binding specialization with different affinities for host biogenic amines [32,35,36], eicosanoids [35,37,38], or nucleosides and nucleotides in the case of a *Culex quinquefasciatus* D7 protein [33]. Both D7 short forms containing a single-domain and D7 long forms containing two-domains are present in the saliva of mosquitoes [34]. In *Aedes* spp. and *Culex* spp., ligand binding properties appear to be restricted to the D7 long forms. A multifunctional mechanism of ligand binding has been described for two-domain D7 proteins in *Ae. aegypti* and *Cx. quinquefasciatus*: The N-terminal domain binds cysteinyl leukotrienes, while the C-terminal domain shows high affinity to biogenic amines such as norepinephrine, serotonin, and histamine [33,35,39].

Although not structurally related, both biogenic amines and eicosanoids act as hemostasis agonists. Among biogenic amines, the catecholamines norepinephrine, epinephrine, and tryptamine stimulate vasoconstriction [15]. Serotonin and histamine induce pain and itch and increase vascular permeability [40]. In addition, serotonin is a weak platelet aggregation agonist. The eicosanoid leukotriene B_4_ (LTB_4_) acts as a potent chemotactic agent of polymorphonuclear cells and cysteinyl leukotrienes C4 (LTC_4_), D4 (LTD_4_), and E4 (LTE_4_), are involved in inflammatory reactions. They also activate vasoconstriction, edema formation, and capillary leakage [41]. Here, we report the characterization of a D7 long form protein from *Ae. albopictus* saliva: AlboD7L1. We have cloned, expressed, and purified AlboD7L1 recombinant protein and show its binding affinities for biogenic amines and eicosanoids. We also demonstrate the functional consequence of scavenging these hemostasis and inflammatory agonists: Biogenic amine binding results in platelet aggregation inhibition whereas leukotriene binding prevents leukocyte recruitment in vivo. This work highlights the role of AlboD7L1 in blood feeding by scavenging pro-hemostatic and immune mediators at the bite site. Future perspectives will focus on investigating the role of this protein in blood feeding and pathogen transmission in vivo through the creation of AlboD7L1 knockout mosquito lines generated by CRISPR/Cas9.

## 2. Materials and Methods

### 2.1. Sequence Alignment

Nucleotide and amino acid sequences of D7 protein genes from the *Ae. albopictus* mosquitoes sialotranscriptome and *Ae. aegypti* genome (AaegL5) were retrieved from the NCBI databases. Multiple alignments were obtained by Clustal Omega [42], using as input the amino acids without signal peptide predicted by SignalP program [43]. Alignment results were refined using BoxShade server [44] and converted to rich text files for figure annotation.

### 2.2. Cloning, Expression, and Purification of AlboDL71

Synthetic AlboD7L1 gene was subcloned into pET-17b (Bio Basic Inc., Markham, ON, Canada) for expression in *Escherichia coli* BL21 (DE3) pLysS cells (Invitrogen, San Diego, CA, USA). Protein expression was carried out as previously described [33]. Inclusion bodies were refolded using 300 mM arginine, 50 mM Tris, pH 8.0. AlboD7L1 recombinant protein was purified, first by size exclusion chromatography on a HiPrep 16/60, followed by cation exchange chromatography using Mono S 5/50 GL column (GE Healthcare Life Science, Piscataway, NJ, USA). A final analytical size exclusion chromatography was performed using a Superdex 200 10/300 GL column (GE Healthcare Life Science, Piscataway, NJ, USA). AlboD7L1 purified protein was separated in NuPAGE 4–12% Bis-Tris Protein Gels (Life Technologies, Carlsbad, CA, USA) and visualized by Coomassie stain. Protein identity was confirmed by Edman degradation at the Research Technologies Branch, NIAID, NIH.

### 2.3. Isothermal Titration Calorimetry

To identify the potential biological function of AlboD7L1, isothermal titration calorimetry (ITC) was used to determine its binding affinities to putative ligands that are known to affect inflammation and hemostasis (biogenic amines, leukotrienes, and thromboxane A_2_) [15,40,41]. Measurements were performed on a MicroCal VP-ITC (Malvern Panalytical, Westborough, MA, USA) at 30 °C and stir speed at 286 rpm. Possible candidate ligands used were six biogenic amines (dopamine, norepinephrine, epinephrine, histamine, tryptamine, and serotonin), five eicosanoid bioactive lipids (LTB_4_, LTC_4_, LTD_4_, LTE_4_, and the thromboxane A_2_ analog U-46619). Protein and ligands were diluted in Tris-buffered saline (TBS-20 mM Tris-HCl, 150 mM NaCl, pH 7.4) and degassed under vacuum for 5 min using a MicroCal ThermoVac (Malvern Panalytical, Westborough, MA, USA). The AlboD7L1 concentrations were 2 μM, 3 μM, or 5 μM, and ligand concentrations were prepared at 10-fold the protein concentration (20 μM, 30 μM, or 50 μM), depending on protein/ligand combination for optimal ITC performance. For eicosanoid ligands, the solvent was first evaporated under a stream of nitrogen gas prior to dissolving in TBS, vortexed, and sonicated for 10 min (Branson 1510, Branson Ultrasonics, Danbury, CT, USA) to ensure dissolution. The protein sample volume in the cell was 1.4 mL, and ligand injections were delivered in 10 μL volume in a total of 31 injections. The data was analyzed using the MicroCal PEAQ-ITC analysis software v 1.21 (Malvern Panalytical, Westborough, MA, USA) by fitting to a single-site binding model to obtain the stoichiometry (N), the dissociation constant (K_D_), the enthalpy of binding (ΔH), and the entropy change (ΔS°) at a constant temperature (T). Because N values were close to 1, as previously described for other salivary D7 proteins [32,33,35,37,38], and as predicted by the structure model, the ITC data were refitted with a fixed N = 1 value.

### 2.4. AlboD7L1 Structure Modell Prediction

A model of the AlboD7L1 structure was generated with I-TASSER software (version 5.1 Zhang Lab, University of Michigan, Ann Arbor, MI, USA) [45] using the mature amino acid sequence as a query. This software predicts the secondary and tertiary structures based on the similarity of other proteins whose structure has been solved. Structural figures and hydrophobicity surface analysis were done using UCSF Chimera (Resource for Biocomputing, Visualization, and Informatics at the University of California, San Francisco, CA, USA) [46].

### 2.5. Ex Vivo Platelet Aggregation Assays

Platelet rich plasma was obtained from normal healthy donors on the NCI IRB approved NIH protocol 99-CC-0168, “Collection and Distribution of Blood Components from Healthy Donors for In Vitro Research Use.” Blood donors provided written informed consent, and platelets were de-identified prior to distribution. Platelet aggregation was measured using a light transmission aggregometer (Chrono-Log Corporation, Havertown, PA, USA). Briefly, 300 μL of platelet rich plasma, diluted to approximately 2.5 × 10^5^ platelets/μL in HEPES-Tyrode’s buffer (137 mM NaCl, 27 mM KCl, 12 mM NaHCO_3_, 0.34 mM sodium phosphate monobasic, 1 mM MgCl_2_, 2.9 mM KCl, 5 mM HEPES, 5 mM glucose, 1% BSA, 0.03 mM EDTA, pH 7.4) were pre-stirred in the aggregometer for 1 min to monitor pre-aggregation effects. Recombinant AlboD7L1 or Tyrode’s buffer as negative control were added to the platelet rich plasma before adding the agonists. Aggregation agonists used in our studies included serotonin, epinephrine, collagen type I fibrils from equine tendons, U-46619, ADP or combination of agonists. Their concentrations are specified in the figure or figure captions. Technical duplicates were performed. For experiments shown in Figure 6 a Chrono-Log aggregometer model 500-CA was used while experiments shown in Supplementary Figure S3 were done in a Chrono-Log model 700.

### 2.6. Animals and In Vivo Leukocyte Recruitment Experiment

Female C3H/HeJ mice, 6–10 weeks old, were purchased from Charles River Laboratories (Germantown, MD, USA) and maintained at the animal facility at the Laboratory of Malaria and Vector Research (Rockville, MD, USA). All procedures were performed in accordance with the animal study protocol approved by the NIAID Animal Care and Use Committee (ASP#: LMVR3). Mice received an i.v. inoculation of 200 μL of PBS or AlboD7L1 (100 μg/kg), and 10 min later each mouse was i.p. injected with 200 μg of β-glucan from *Saccharomyces cerevisiae* (Calbiochem-EMD Millipore, Billerica, MA, USA). The control group consisted of mice receiving the same volume of PBS through the same routes. After 24 h, mice were euthanized, and the cells from the peritoneal cavity were harvested by injection of 3 mL of PBS containing 0.38% sodium citrate. Cells recruited to the peritoneal cavity were concentrated using a cyto centrifuge (StatSpin Cytofuge 2, Beckman Coulter, Indianapolis, IN, USA) and sample preparations were stained with a three-Step Stain kit (Thermo Fisher Scientific Inc., Waltham, MA, USA). Total cells recruited to the peritoneal cavity were determined in a Neubauer chamber. Differential counts were performed under light microscopy in cytocentrifuge preparations (StatSpin Cytofuge 2, Beckman Coulter, Indianapolis, IN, USA) stained with a three-Step Stain kit (Thermo Fisher Scientific Inc., Waltham, MA, USA). Briefly, two-hundred cells were counted and the percentage of neutrophils, eosinophils, mast cells, and mononuclear cells present on each sample was visually evaluated in a blinded fashion (the operator did not know the group each sample came from) and the number of each cell type was calculated as previously described [47]. The experiment was performed twice (n = 3 mice/group) with similar results and data. Results are shown as mean ± standard error of the mean (SEM) of one experiment. One-way analysis of variance (ANOVA), followed by Bonferroni post hoc test, was used to compare the groups. A *p*-value of 0.05 or less was considered statistically significant.

## 3. Results

### 3.1. Initial Characterization of AlboD7L1

**Figure 1:**
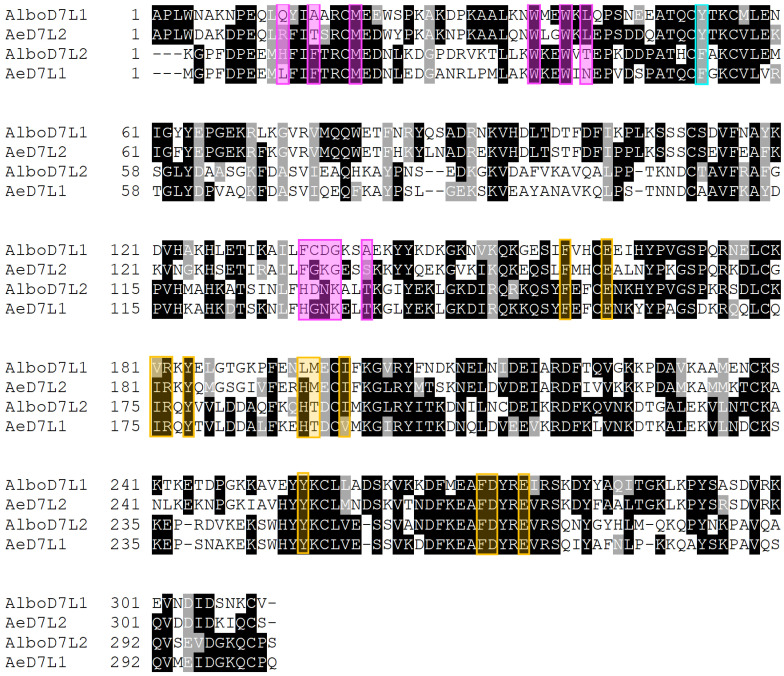
Sequence alignment of AlboD7 and AeD7 salivary proteins. Comparison of AlboD7 and AeD7 long form salivary proteins: AlboD7L1 (GenBank: AAV90665.1), AeD7L2 (GenBank: AAL16049), AlboD7L2 (GenBank: AAV90666.1), and AeD7L1 (GenBank: AAA29347). Sequences without a signal peptide were aligned with Clustal Omega and refined using the BoxShade server. Black background shading represents identical amino acids, while grey shading shows similar amino acids. The magenta boxes show the predicted amino acids involved in the U-46619, thromboxane A_2_ analog; the cyan box indicates the Tyr-52 predicted to be involved in the TxA_2_ binding, based on the solved crystal structure of the AnSt-D7L1 protein [37]; the yellow boxes reflect the amino acids involved in the norepinephrine binding according to the crystal structure obtained from AeD7L1 bound to norepinephrine [35].

Sequence comparisons between salivary D7 long form proteins from *Ae. albopictus* and *Ae. aegypti* suggest that many of the residues involved in the binding of ligands are conserved. Like *Ae. aegypti*, *Ae. albopictus* has two D7 long forms in its saliva (AlboD7L1 and AlboD7L2). Interestingly, AlboD7L1 sequence is very similar to AeD7L2 with 66.47% identity (BLASTp *e*-value: 7 × 10^−171^); whereas AlboD7L2 shares 69.57% identity (BLASTp *e*-value: 2 × 10^−167^) with AeD7L1 (Figure 1).

**Figure 2:**
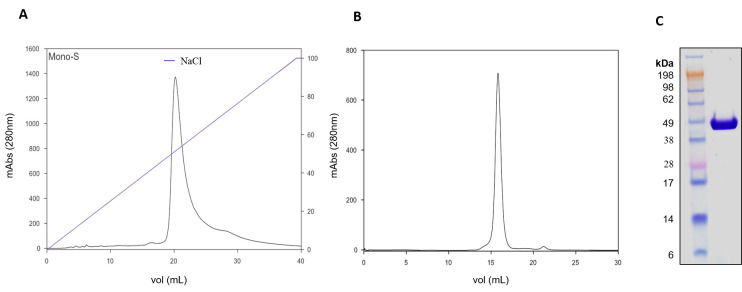
Purification of recombinant AlboD7L1. (**A**) Purification of AlboD7L1 by cation-exchange chromatography using a MonoS 5/50 GL column. Gradient of NaCl (%) is indicated by the blue line. (**B**) Purification of AlboD7L1 by size exclusion chromatography using a Superdex 200 Increase 10/300 GL column. (**C**) Coomassie-stained NuPAGE 4–12% Bis-Tris gel electrophoresis of recombinant protein AlboD7L1. SeeBlue Plus2 Pre-stained protein ladder was used as protein standards.

For binding assays, recombinant AlboD7L1 was expressed in *E. coli* and purified by ion-exchange and size-exclusion chromatography as described in Materials and Methods (Figure 2A,B). The identity of purified recombinant protein was confirmed by N-terminal sequencing and visualized as a single band by Coomassie-staining gel electrophoresis (Figure 2C).

### 3.2. Binding Properties of AlboD7L1

**Figure 3:**
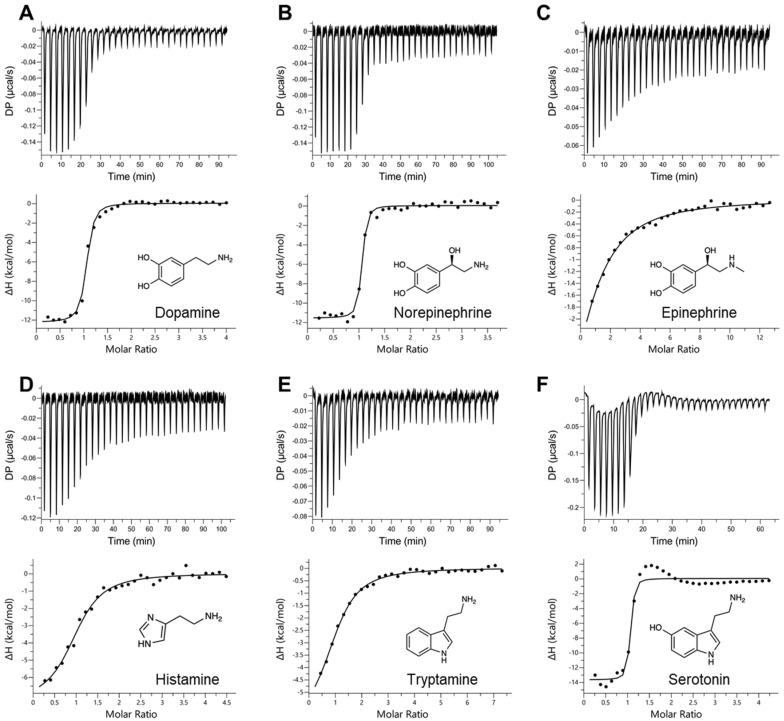
Binding of AlboD7L1 to biogenic amines by isothermal titration calorimetry. Binding experiments were performed on a VP-ITC microcalorimeter. The upper curve in each panel shows the measured heat for each injection, while the lower graph shows the enthalpies for each injection and the fit to a single-site binding model for calculation of thermodynamic parameters. Binding of AlboD7L1 to dopamine (**A**), norepinephrine (**B**), epinephrine (**C**), histamine (**D**), tryptamine (**E**), and serotonin (**F**). The insets show the names and chemical formulas for these compounds.

**Figure 4:**
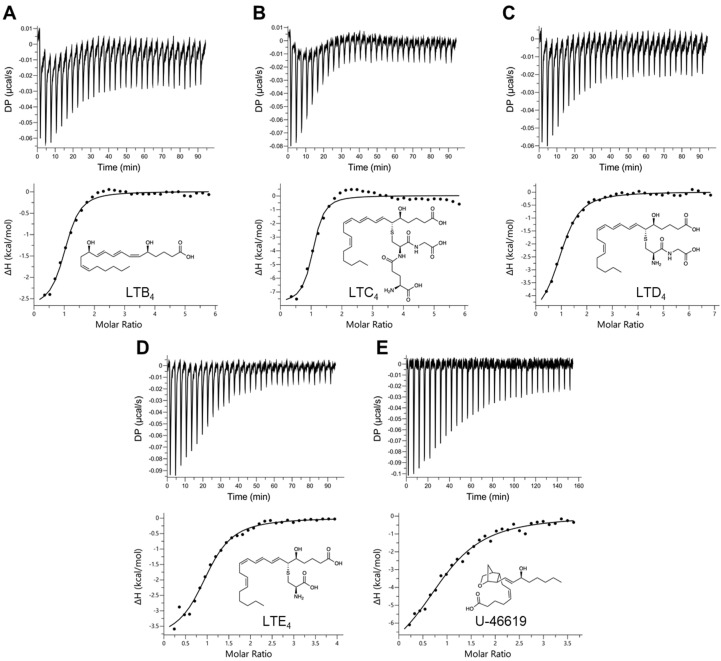
Binding of AlboD7L1 to biolipids by isothermal titration calorimetry. Binding experiments were performed on a VP-ITC microcalorimeter. The upper curve in each panel shows the measured heat for each injection, while the lower graph shows the enthalpies for each injection and the fit to a single-site binding model for calculation of thermodynamic parameters. Binding of AlboD7L1 to LTB_4_ (**A**), LTC_4_ (**B**), LTD_4_ (**C**), LTE_4_ (**D**), and U-46619 (**E**). The insets show the names and chemical formulas for these compounds.

In order to assess the binding affinities of the selected ligands to AlboD7L1, ITC experiments were performed to determine the dissociation constant (K_D_) of each protein ligand combination. As shown in Table 1, Figure 3 and Figure 4 and Supplementary Figure S1, AlboD7L1 was able to bind all ligands tested (dopamine, serotonin, histamine, tryptamine, epinephrine, norepinephrine, leukotrienes B_4_, C_4_, D_4_, E_4_, and U-46619). AlboD7L1 also binds a single molecule of each ligand at varying affinities (stoichiometry of 1). It had a high affinity to dopamine (K_D_ = 11.0 ± 2.19 nM). Overall, the binding affinities were highest to the biogenic amines (Figure 3), in particular norepinephrine (K_D_ = 3.67 ± 0.994 nM) and serotonin (K_D_ = 4.51 ± 2.66 nM). AlboD7L1 had lower binding to histamine (K_D_ = 278 ± 66.2 nM) and tryptamine (K_D_ = 570 ± 97.7 nM), similar to AeD7L2 affinities for these ligands. A lower affinity to epinephrine (K_D_ = 4110 ± 3330 nM) was calculated. AlboD7L1 was also able to bind the thromboxane A_2_ analog U-46619 (K_D_ = 946 ± 269 nM, Figure 4), for which AeD7L1 showed no binding. AlboD7L1 showed a similar binding profile for leukotrienes, as previously reported for AeD7L1. As shown in Figure 4, the highest binding affinity of AlboD7L1 was to LTC_4_ (K_D_ = 67.7 ± 22.4 nM), with lowest binding affinities to LTB_4_ (K_D_ = 342 ± 65.0 nM), LTD_4_ (K_D_ = 332 ± 48.3 nM), and LTE_4_ (K_D_ = 567 ± 115 nM).

### 3.3. AlboD7L1 Structure Modeling

**Figure 5:**
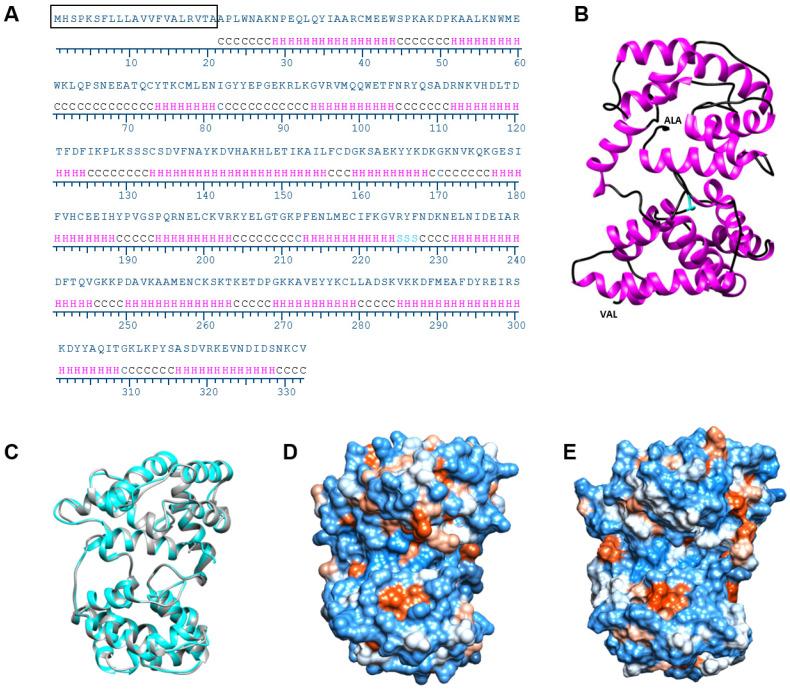
Structural model of AlboD7L1. (**A**) Secondary structure prediction of AlboD7L1 using I-TASSER software. Prediction for coils (C), helixes (H), and strands (S) are indicated. (**B**) Tertiary structure model of AlboD7L1 predicted by I-TASSER and visualized in Chimera software. N-terminal is indicated by ALA-1, and C-terminal is shown as VAL-311 of the mature protein sequence. Coils and helixes are depicted in black and magenta, respectively. The three amino acids predicted to form a strand are represented in cyan. (**C**) Superposition of AeD7L1 (PDB ID: 3DXL, shown in grey) and protein structure model of AlboD7L1, represented in cyan, shows an overall similar helix structure. (**D**) Hydrophobicity surface potential of AeD7L1 (PDB ID: 3DXL) generated by Chimera software with blue being positive and red being negative. (**E**) Hydrophobicity surface potential of the AlboD7L1 model.

In silico analysis using the I-TASSER software identified AeD7L1 (PDB ID: 3DXL) as the most similar protein to AlboD7L1 among those proteins whose crystal structures have been solved and are available in the PDB database [48]. AlboD7L1 is predicted to contain 15 alpha helixes (Figure 5A,B), similarly to other D7 proteins, including AeD7L1 and Anst-D7L1 (PDB ID: 3NGV). Although AlboD7L1 and AeD7L1 share 35% identity at the amino acid level (BLASTp *e*-value: 1 × 10^−55^, Figure 1), superposition of the AlboD7L1 model with AeD7L1 solved structure showed that both proteins are structurally similar (Figure 5C) and show a similar hydrophobicity patterns (Figure 5D,E). The protein model predicts that AlboD7L1 might bind biolipids through its N-terminal domain and biogenic amines through its C-terminal domain (Supplementary Figure S2), as shown in AeD7L1 and CxD7L2 [33,35].

### 3.4. AlboD7L1 Inhibits Platelet Aggregation

**Figure 6:**
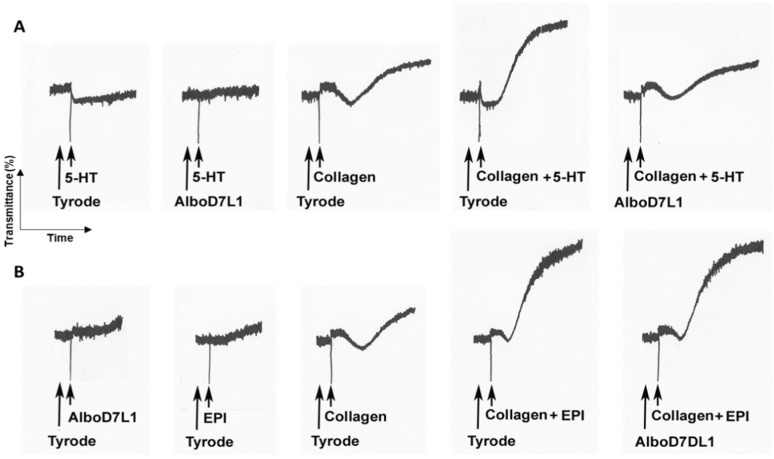
AlboD7L1 inhibits pro-aggregatory effects of serotonin, but not epinephrine. (**A**) AlboD7L1 inhibits 5-HT-induced shape change and 5-HT-induced potentiation of platelet aggregation triggered by collagen. Platelets were incubated with Tyrode (vehicle) or AlboD7L1 (1 μM), followed by the addition of serotonin (5-HT, 0.8 μM) alone or plus collagen (0.75 μg/mL), as indicated. From *left* to *right*, traces show shape change with 5-HT alone, inhibition of 5-HT-induced shape change in the presence of AlboD7L1, potentiation of the collagen response by co-addition of 5-HT resulting in a full aggregation response, and loss of potentiation by 5-HT in the presence of AlboD7L1. (**B**) AlboD7L1 does not inhibit epinephrine-mediated potentiation of platelet aggregation induced by collagen. Platelets were incubated with Tyrode (vehicle) or AlboD7L1 (1 μM), followed by the addition of epinephrine (EPI 0.8 μM) plus collagen (0.75 μg/mL), as indicated. From *left* to *right*, traces show no response of platelets to AlboD7L1 and epinephrine, shape change induced by collagen alone, potentiation of the collagen response by co-addition of epinephrine resulting in a full aggregation response, and no loss of potentiation by epinephrine in the presence of AlboD7L1. A Chrono-Log aggregometer model 500-CA was used.

AlboD7L1 binds platelet agonists such as serotonin, epinephrine and U-46619. Therefore, we hypothesize whether AlboD7L1 functions as a potential platelet aggregation inhibitor. Serotonin acts as a potentiator of platelet agonists such as ADP or collagen. Alone, serotonin can induce platelet shape change; however, in the absence of a more potent agonist, the platelets eventually disaggregate (Figure 6A). AlboD7L1 tightly binds to serotonin as shown in Figure 3. Therefore, the initiation of aggregation produced by serotonin was completely abolished in the presence of AlboD7L1 (Figure 6A). Co-administration of serotonin and low dose of collagen (0.75 μg/mL) as platelet agonists produced a full aggregation response. When platelets were incubated with AlboD7L1, the synergistic effect of serotonin and collagen in platelet aggregation was abolished. Epinephrine also potentiated aggregation initiated by low doses of collagen (Figure 6B). However, AlboD7L1 did not prevent epinephrine and collagen’s synergistic effect as platelet aggregation agonists (Figure 6B).

When platelet aggregation was initiated with high doses of the thromboxane A_2_ analog U-46619, AlboD7L1 did not have any effect in inhibiting aggregation. However, when lower U-46619 doses were used, platelets started to aggregate and as the agonist was being sequestered by AlboD7L1 platelets disaggregated (Supplementary Figure S3A). Exposure of circulating platelets to collagen from the subendothelial matrix or thrombin leads to the formation of a platelet monolayer that supports subsequent adhesion of activated platelets to one another. At low concentrations of collagen (2 μg/mL), TxA_2_ plays an important role in the extension and amplification step of the platelet plug formation. Our experiments strongly indicate that the potent inhibitory effect on platelet aggregation observed for AlboD7L1 is due to its binding to U-46619 and presumably TxA2 (Supplementary Figure S3B). AlboD7L1 did not interfere with platelet aggregation induced by high doses of collagen (10 μg/mL) which results in full platelet aggregation independently of secondary mediators (Supplementary Figure S3B). AlboD7L1 neither prevented aggregation initiated by ADP (Supplementary Figure S3C).

### 3.5. AlboD7L1 Prevents Leukocyte Recruitment In Vivo

**Figure 7:**
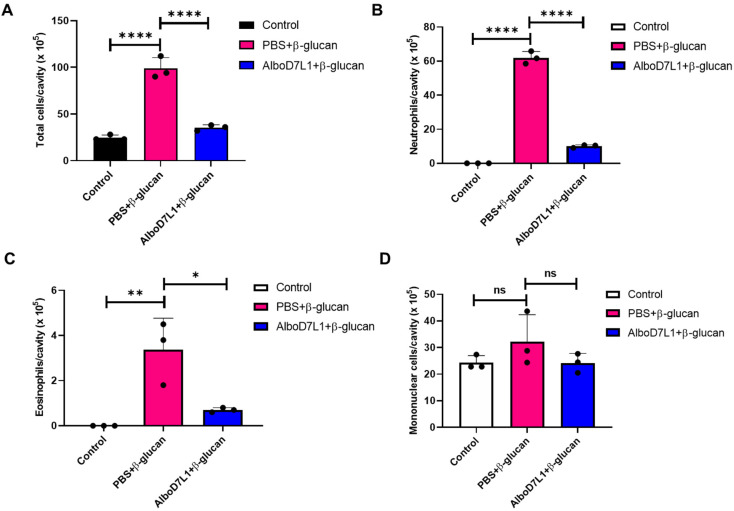
AlboD7L1 prevents cell recruitment caused by *Saccharomyces cerevisiae* β-glucan to the peritoneal cavity in mice. (**A**) Total cell number, (**B**) neutrophils, (**C**) eosinophils, and (**D**) mononuclear cells were collected from the peritoneal cavity of mice after i.v. pretreatment with PBS or AlboD7L1 (100 μg/kg), followed by a i.p. injection of 200 μg of β-glucan from S. cerevisiae. Cell numbers are expressed as cells/cavity (×105). Results are shown as mean ± standard error of the mean (SEM). One-way analysis of variance (ANOVA) followed by Bonferroni post hoc test was used to compare the groups (*p ≤ 0.05; **p ≤ 0.01; ****p ≤ 0.0001).

To evaluate the in vivo effect of biolipid binding by AlboD7L1, a model of leukotriene-dependent peritonitis induced by β-glucan from *Saccharomyces cerevisiae* was employed in C3H/HeJ mice. The influx of mononuclear cells, neutrophils, and eosinophils into the peritoneal cavity was evaluated in mice pretreated with PBS or AlboD7L1 and then inoculated with β-glucan. As expected, the injection of β-glucan induced a robust cell influx to the peritoneal cavity, notably of neutrophils and eosinophils, as compared to the control group (*p* ≤ 0.0001). Pretreatment of mice with AlboD7L1 prevented the inflammation due to β-glucan in this body compartment (Figure 7A, *p* ≤ 0.0001). The neutrophil and eosinophil inflammation triggered by this stimulus is known to be dependent of leukotrienes [49]. Specifically, migration of neutrophils and eosinophils due to β-glucan was significantly inhibited in the AlboD7L1 pre-treated group (Figure 7B, *p* ≤ 0.0001 and Figure 7C, *p* ≤ 0.05, respectively). On the other hand, mononuclear cell numbers were not significantly altered by β-glucan stimulus and AlboD7L1 did not change this phenotype (Figure 7D).

## 4. Discussion

Salivary proteins are injected into the bite site to assist mosquito blood feed. These bioactive components counteract host hemostatic and immune response mediators. Here, we characterized AlboD7L1, one of the most abundant proteins of the salivary glands of the Asian tiger mosquito, *Ae. albopictus*. The biochemical and functional activities of this protein indicate that AlboD7L1 plays an important role in mosquito blood feeding.

Vertebrate hosts respond to an arthropod bite through several mechanisms. Mast cells, platelets and peripheral nerve tissues around the bite site produce biogenic amines and eicosanoids to induce coagulation, platelet aggregation, vasoconstriction, and cause pain and itch responses. These response mechanisms to prevent blood loss and alert the host of the presence of the feeder can be detrimental in obtaining a successful blood meal. In this study, we tested several mediators of host hemostasis and found that AlboD7L1 is capable of binding to all of them at varying affinities. Similar to AeD7L1, AlboD7L1 had the highest affinity to biogenic amine (serotonin and norepinephrine). Serotonin, norepinephrine, epinephrine, and tryptamine stimulate vasoconstriction and reduce the blood flow to the injury site [15,50]. We detected binding to tryptamine, a natural vasoconstrictor similar to serotonin [50]. This is the first time tryptamine binding is tested with a D7 protein. Further studies are needed to understand the role of tryptamine binding in host hemostasis prevention. AlboD7L1 also had high affinity towards dopamine. Although the exact role of dopamine in the vertebrate host skin is still largely unknown, some evidence suggests that it is involved in modulating wound healing [51]. The fact that AlboD7L1 binds both histamine and serotonin suggests that this salivary protein might reduce the itching, swelling, and pain felt at the bite site, as a result of increased vascular permeability and infiltration of monocytes induced by these mediators [40,52]. Given the K_D_ values between histamine and its physiological receptors are in the nM range [53], AlboD7L1 would be a good competitor for scavenging histamine.

Overall, the binding affinities to the eicosanoids were lower than biogenic amines. Of the leukotrienes, AlboD7L1 had the highest affinity to LTC_4_. Leukotrienes are released by mast cells and act quickly to induce pain, swelling, itching, and erythema [54,55]. The thromboxane A_2_ (TXA_2_) analog, U-46619, is a potent vasoconstrictor, similar to serotonin and epinephrine. Much like epinephrine, AlboD7L1 had a significantly reduced binding affinity to U-46619. There is a great deal of redundancy in the vertebrate host response to skin injury, and this perhaps explains the coevolution of D7 proteins to bind so many diverse molecules. *Aedes albopictus* expresses six D7 proteins, two long form and four short form [23].

Sequence comparisons between AlboD7L1 and *Ae. aegypti* D7 long form proteins showed that most of the residues involved in ligand binding are conserved. AlboD7L1 has the highest similarity to AeD7L2, which corresponds well with the positive binding to U-46619 [39]. *Anopheles stephensi* AnSt-D7L1 also binds U-46619; the solved crystal structure of the protein with the bound ligand demonstrated that U-46619 is stabilized by hydrogen bonding interactions of the omega-5 hydroxyl group with the phenolic hydroxyl group of Tyr-52 [37]. Tyr-52 is present in both AlboD7L1 and AeD7L2; however, it is absent in AeD7L1, which does not bind U-46619 [35]. The structural model of AlboD7L1 predicts a similar structure and hydrophobicity pattern to AeD7L1, the only salivary D7 protein from *Ae. aegypti* that has been solved so far [35]. AlboD7L1 might bind biolipids through its N-terminal domain and biogenic amines through its C-terminal domain, in a similar manner of AeD7L1 and CxD7L2 [33,35].

AlboD7L1 tightly binds serotonin, which is released from platelet dense bodies upon activation. Besides inducing constriction of injured blood vessels, serotonin and epinephrine enhance platelet aggregation to minimize blood loss [40]. Platelets play a pivotal role in primary hemostasis. Exposure of circulating platelets to collagen from the subendothelial matrix or thrombin leads to the formation of a platelet monolayer that supports subsequent adhesion of activated platelets to one another [15,56]. Serotonin and epinephrine act as weak platelet agonists alone, but more importantly, they reduce the threshold concentrations of other agonists, including collagen, for platelet aggregation. AlboD7L1 scavenges serotonin and prevents its potentiating effect to collagen, resulting in an inhibition of platelet aggregation, as previously observed for *Cx. quinquefasciatus* D7L2 and the biogenic amine-binding protein from the triatomine *Rhodnius prolixus* [33,57]. However, we did not observe any loss of the potentiation effect of platelet aggregation induced by the combination of collagen and epinephrine in the presence of AlboD7L1. This lack of inhibition might be a result of a lower binding affinity of AlboD7L1 to epinephrine. When platelets are activated, TXA_2_ is synthesized from arachidonic acid released from platelet membrane phospholipids. TXA_2_ is an unstable compound and cannot be evaluated directly as a platelet aggregation agonist ex vivo. We used U-46619, the stable analog of TXA_2_ and widely accepted for platelet aggregation studies. AeD7L1 inhibits platelet aggregation induced by low doses of U-46619 most probably due to the AlboD7L1′s low binding affinity to this agonist. TxA_2_ and ADP play an important role on the extension and amplification step of the platelet plug formation. AlboD7L1 did not prevent platelet aggregation initiated by ADP, as most probably this protein does not bind this mediator. ADP binding has been demonstrated only for *Cx. quinquefasciatus* D7L1 [33]. Besides, the residues of CxD7L1 involved in ADP-binding are not conserved in AlboD7L1. Or results show that AlboD7L1 prevents aggregation mediated by low doses of collagen (2 μg/mL) without having any effect on aggregation initiated by high doses of collagen (10 μg/mL). At high concentrations, collagen acts as a strong agonist of the GPVI receptor on platelet surface, which induces platelet aggregation in an independent manner of ADP or TXA_2_ [56]. At low concentrations of collagen, ADP and TXA_2_ play an important role on the extension and amplification step of the platelet plug formation. Upon platelet activation, mediators secreted by platelets bind to G protein-coupled receptors in platelet membranes, rapidly amplifying the aggregation signal in a positive feedback response [58].

The mouse model of β-glucan induced peritonitis allowed us to study the effect of AlboD7L1 in preventing cell recruitment mediated by leukotrienes. Many immune cells express receptors capable of recognizing β-glucan in its various forms. For example, the binding of β-glucan with integrins CD11b/CD18 (Mac-1), present on neutrophils and mononuclear cell surface, induces the activation of the enzyme 5-lipoxygenase and the concomitant release of LTB_4_ contributing to the amplification of the inflammatory response [59,60]. In fact, β-glucan of *Histoplasma capsulatum* is involved in leukocyte recruitment to the site of inoculation, and the leukotrienes are the main chemoattractant involved in early neutrophil and eosinophil migration (6 to 24 h), and late mononuclear cell migration (48 to 72 h) in this model [47,49]. AlboD7L1 pretreatment significantly reduced cell infiltration into the peritoneal cavity of mice when compared to control animals. These results suggest that the effect of AlboD7L1 on the recruitment of neutrophils and eosinophils induced by the β-glucan from *S. cerevisiae*, is due to its inhibitory effect on leukotrienes, especially LTB_4_ which is a potent stimulant of leukocyte functions including the chemotaxis, chemokinesis, and aggregation of polymorphonuclear leukocytes. Moreover, LTB_4_, LTD_4_, LTC_4_, and LTE_4_ are chemoattractant for eosinophils [49]. Here, we have shown that leukotriene binding, a shared feature between AlboD7L1 and *Ae. aegypti* D7 proteins [35,39], leads to the inhibition of leukocyte recruitment, an ability that might be beneficial for mosquito blood feeding. Both neutrophils and eosinophils secrete pro-inflammatory cytokines and hemostasis mediators including cysLTs, prostaglandins, and platelet activation factor [61,62]. Neutrophils are the first leukocytes to be recruited to an inflammatory site such as those caused by blood feeding arthropods. Recent research has linked hemostasis with inflammation based on the effects of neutrophil extracellular traps (NETs) on platelets and coagulation [63]. NETs promote platelet aggregation, secretion of ATP and ADP, and increased expression of CD62L and phosphatidylserine (PS) on the surface of the platelets [64]. Besides, NETs can initiate coagulation via thrombin activation [63]. In our experiments, we did not observe an effect of AlboD7L1 on mononuclear cell recruitment. It is possible that it is not the salivary protein that has no effect on mononuclear cells, but rather the beta-glucan that did not increase the recruitment of these cells. At least, no significant increases were seen relatively to control, after beta-glucan administration. Our results agree with previous studies that used this peritonitis model [47].

We demonstrated that the binding of AlboD7L1 to hemostatic mediators results in inhibition of platelet aggregation in ex vivo experiments. Previous work on D7 proteins from *Cx. quinquefasciatus* has correlated the platelet aggregation inhibition activity observed ex vivo with an anti-hemostatic biological activity in vivo in a mouse model of tail bleeding [33]. We also show that AlboD7L1 binds other well-known mediators of itch, pain, and vasoconstriction. In previous work, we and others have shown that salivary proteins binding to these mediators induce a strong vasodilation in ex vivo experiments using contractility assays [39] and reviewed in [16,65]. Thus, we hypothesize that AlboD7L1 might prevent hemostasis in vivo to facilitate blood feeding. However, a more in-depth study will be necessary to experimentally demonstrate the in vivo activity of AlboD7L1.

## 5. Conclusions

In conclusion, we characterized a novel D7 long form protein from *Ae. albopictus* saliva at the biochemical and functional level. We determined the binding capabilities of AlboD7L1 which were similar to the *Ae. aegypti* D7 salivary proteins. Our structural predicted model suggests that AlboD7L1 binds biogenic amines and biolipids independently through the C-terminal and N-terminal domain, respectively. Moreover, we demonstrated the AlboD7L1 role in inhibiting both platelet aggregation and cell recruitment of polymorphonuclear cells. These anti-hemostatic and anti-inflammatory properties might help blood feeding in mosquitoes. Importantly, this work reinforces the relevance of the D7 family of salivary proteins, which are among the most abundant proteins in mosquito saliva. Future work will investigate the role of this protein in blood feeding and pathogen transmission in vivo through the creation of AlboD7L1 knockout mosquito lines generated by CRISPR/Cas9.

## Figures and Tables

**Table 1:** Binding affinities of AlboD7L1 with biogenic amine and eicosanoid ligands measured by isothermal titration calorimetry (ITC).

Ligand	K_D_ (nM) ± SE	ΔH (kcal/mol) ± SE	−TΔS (kcal/mol)
Dopamine ^†^	11.0 ± 2.19	−12.3 ± 0.197	1.23
Norepinephrine ^†^	3.67 ± 0.994	−11.6 ± 0.186	−0.116
Epinephrine ^†^	4110 ± 3330	−6.83 ± 0.809	−0.637
Histamine ^†^	278 ± 66.2	−7.47 ± 0.378	−1.63
Tryptamine ^†^	570 ± 97.7	−6.43 ± 0.223	−2.23
Serotonin ^‡^	4.51 ± 2.66	−13.7 ± 0.445	2.13
LTB_4_^§^	342 ± 65.0	−2.74 ± 0.085	−6.23
LTC_4_^†^	67.7 ± 22.4	−7.90 ± 0.350	−2.05
LTD_4_^†^	332 ± 48.3	−5.00 ± 0.139	−3.98
LTE_4_^§^	567 ± 115	−3.98 ± 0.180	−4.69
U-46619 ^‡^	946 ± 269	−8.54 ± 0.785	0.189

^†^ Protein concentration in the cell = 2 μM, ligand concentration in the syringe = 20 μM; ^‡^ Protein concentration in the cell = 3 μM, ligand concentration in the syringe = 30 μM; ^§^ Protein concentration in the cell = 5 μM, ligand concentration in the syringe = 50 μM.

## Supplementary Materials

The following are available online at https://www.mdpi.com/2218-273X/10/10/1372/s1, Figure S1: Comparison of binding affinities of several mosquito D7 salivary proteins to biogenic amines and biolipids involved in hemostasis. Figure S2: Binding pockets prediction of AlboD7L1 for eicosanoids and biogenic amines. Figure S3. AlboD7L1 inhibits pro-aggregatory effects of the thromboxane A_2_ analog U-46619, low doses of collagen but not ADP.
