# Supplementary material for: Aedes albopictus D7 Salivary Protein Prevents Host Hemostasis and Inflammation
Source: Biomolecules. 2020 Sep 27;10(10):1372. doi: 10.3390/biom10101372 (PMC7601585; doi:10.3390/biom10101372)
Supplement: Supplementary file 1 [file biomolecules-10-01372-s001.pdf]

<u>**Supplementary figures**</u>

Supplementary Fig. 1.

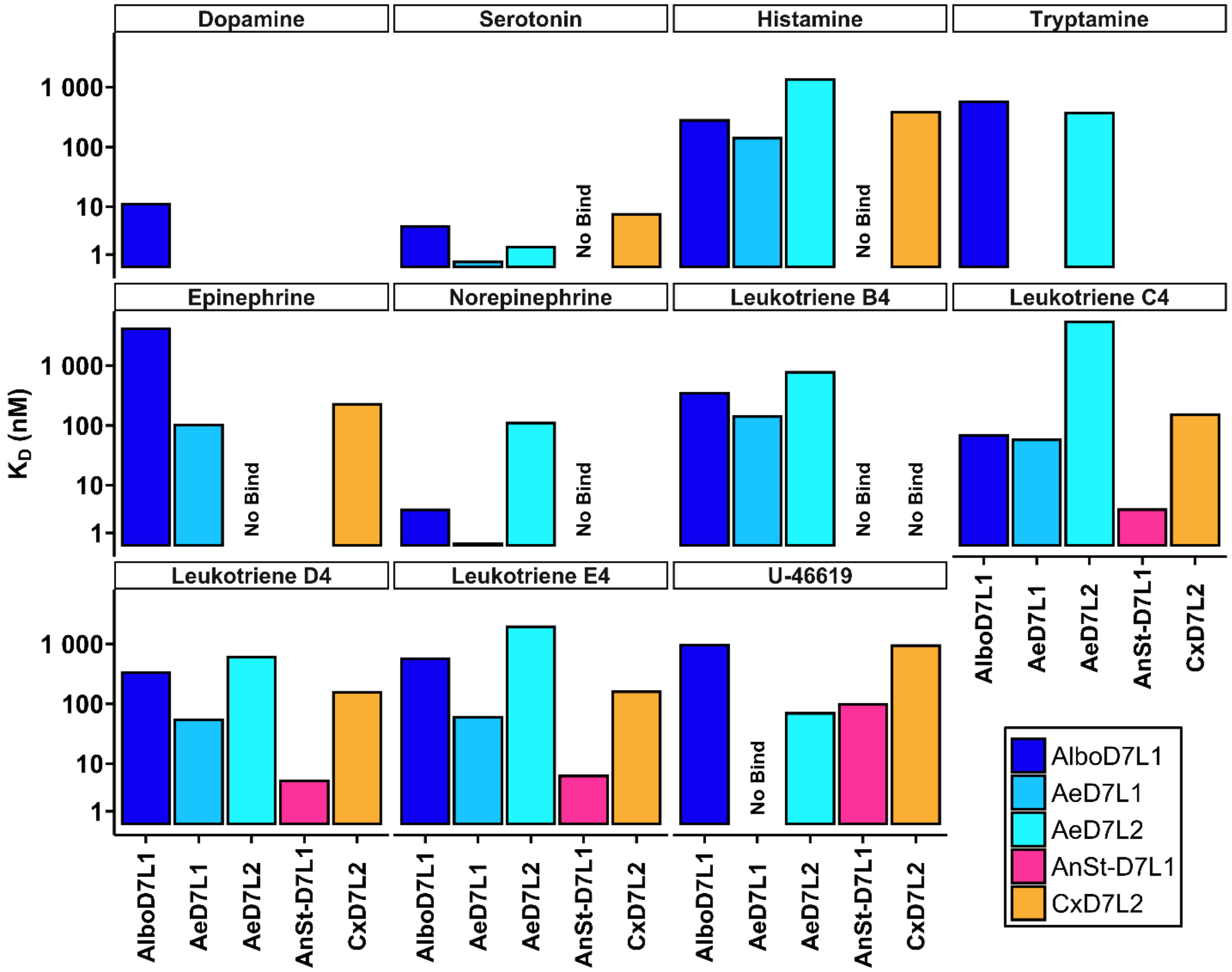

Supplementary Fig. 1. Comparison of binding affinities of several mosquito D7 salivary proteins to biogenic amines and biolipids involved in hemostasis. The equilibrium dissociation constant (KD) of AeD7L1 [1,2], AeD7L2 [3], AlboD7L1 (represented in blue), CxD7L2 [4], and AnSt-D7L1 [5] are reported. A "No bind" designation represents no protein-ligand binding and blank bar space indicates binding affinity has not been tested.

Supplementary Fig. 2.

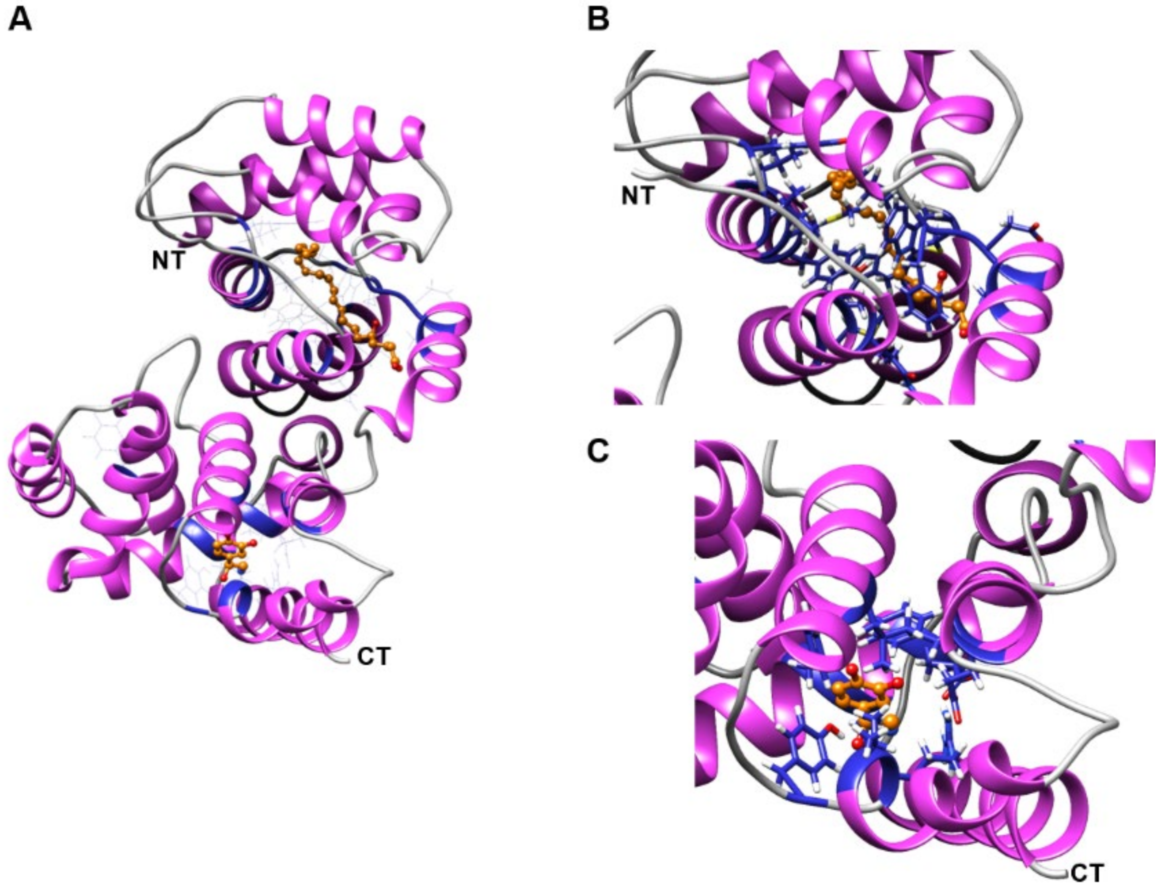

Supplementary Fig. 2. Binding pockets prediction of AlboD7L1 for eicosanoids and biogenic amines. (**A**) Ribbon representation of AlboD7L1 protein structure model shows the potential amino acids involved in leukotriene E_4 and norepinephrine represented in blue and shown as wires. (**B**) Detail of the predicted leukotriene E_4 binding pocket based on similarity with AeD7L1 (PBD ID: 3DZT) that corresponded to the dotted area in Fig. S2. (**C**) Detail of the predicted norepinephrine binding pocket based on similarity with AeD7L1 (PBD ID: 3DYE) that corresponded to the dotted area in Fig S2. Ligands are represented in orange as balls and sticks. N-terminal and C-terminal are labelled as NT and CT, respectively.

Supplementary Fig. 3.

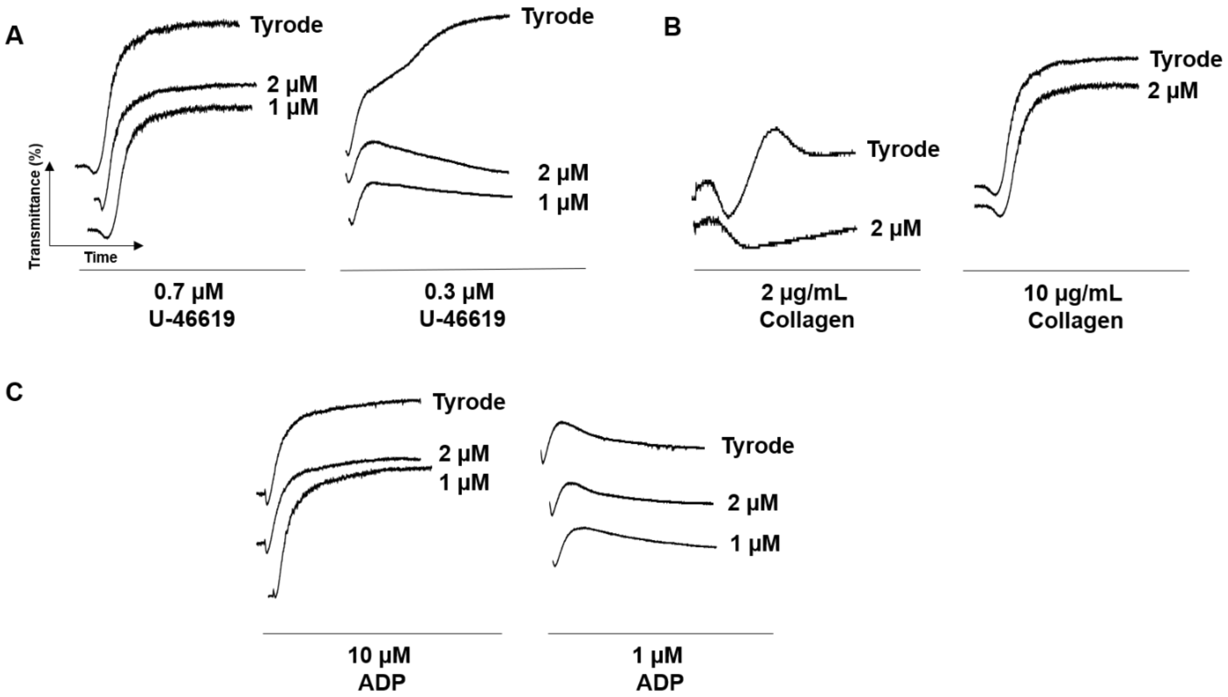

Supplementary Fig. 3. AlboD7L1 inhibits pro-aggregatory effects of the thromboxane A2 analog U-46619, low doses of collagen but not ADP. (**A**) AlboD7L1 does not inhibit high doses of U-46619 while it prevented aggregation triggered by lower doses of U-46619. (**B**) AlboD7L1 inhibits low doses of collagen-mediated aggregation (2 μg/mL) while it failed to prevent aggregation induced by high doses of collagen (10 μg/mL). (**C**) AlboD7L1 did not prevent aggregation induced by ADP. Control samples are indicated as Tyrode (vehicle). A Chrono-Log aggregometer model 700 was used.

References from Supplementary material

1. Calvo, E.; Mans, B.J.; Ribeiro, J.M.; Andersen, J.F. Multifunctionality and mechanism of ligand binding in a mosquito antiinflammatory protein. *Proc Natl Acad Sci U S A* **2009**, *106*, 3728-3733, doi:10.1073/pnas.0813190106.
2. Calvo, E.; Mans, B.J.; Andersen, J.F.; Ribeiro, J.M. Function and evolution of a mosquito salivary protein family. *J Biol Chem* **2006**, *281*, 1935-1942, doi:10.1074/jbc.M510359200.
3. Martin-Martin, I.; Kern, O.; Brooks, S.; Smith, L.A.; Valenzuela-Leon, P.C.; Bonilla, B.; Ackerman, H.; Calvo, E. Biochemical characterization of AeD7L2 and its physiological relevance in blood feeding in the dengue mosquito vector, *Aedes aegypti*. *FEBS J* **2020**, 10.1111/febs.15524, doi:10.1111/febs.15524.
4. Martin-Martin, I.; Paige, A.; Valenzuela Leon, P.C.; Gittis, A.G.; Kern, O.; Bonilla, B.; Chagas, A.C.; Ganesan, S.; Smith, L.B.; Garboczi, D.N., et al. ADP binding by the *Culex quinquefasciatus* mosquito D7 salivary protein enhances blood feeding on mammals. *Nat Commun* **2020**, *11*, 2911, doi:10.1038/s41467-020-16665-z.
5. Alvarenga, P.H.; Francischetti, I.M.; Calvo, E.; Sa-Nunes, A.; Ribeiro, J.M.; Andersen, J.F. The function and three-dimensional structure of a thromboxane A2/cysteinyl leukotriene-binding protein from the saliva of a mosquito vector of the malaria parasite. *PLoS Biol* **2010**, *8*, e1000547, doi:10.1371/journal.pbio.1000547.
